# Thiocarbonyl Derivatives of Natural Chlorins: Synthesis Using Lawesson’s Reagent and a Study of Their Properties

**DOI:** 10.3390/molecules28104215

**Published:** 2023-05-20

**Authors:** Viktor Pogorilyy, Petr Ostroverkhov, Valeria Efimova, Ekaterina Plotnikova, Olga Bezborodova, Ekaterina Diachkova, Yuriy Vasil’ev, Andrei Pankratov, Mikhail Grin

**Affiliations:** 1 Department of Chemistry and Technology of Biologically Active Compounds, Medicinal and Organic Chemistry, Institute of Fine Chemical Technologies, MIREA-Russian Technological University, 86 Vernadsky Avenue, 119571 Moscow, Russiaandreimnioi@yandex.ru (A.P.);; 2 P. Hertsen Moscow Oncology Research Institute—Branch of the National Medical Research Radiological Centre of the Ministry of Health of the Russian Federation, 2nd Botkinsky pr., 3, 125284 Moscow, Russia; 3 Department of Oral Surgery, Borovsky Institute of Dentistry, I.M. Sechenov First Moscow State Medical University (Sechenov University), str Trubetskaya 8\2, 119435 Moscow, Russia; 4 Department of Operative Surgery and Topographic Anatomy, I.M. Sechenov First Moscow State Medical University (Sechenov University), str Trubetskaya 8\2, 119435 Moscow, Russia; y_vasiliev@list.ru

**Keywords:** photodynamic therapy (PDT), photosensitizers (PS), combined chemotherapy and PDT, Lawesson’s reagent (LR), platinum complex, cisplatin

## Abstract

The development of sulfur-containing pharmaceutical compounds is important in the advancement of medicinal chemistry. Photosensitizers (PS) that acquire new properties upon incorporation of sulfur-containing groups or individual sulfur atoms into their structure are not neglected, either. In this work, a synthesis of sulfur-containing derivatives of natural chlorophyll *a* using Lawesson’s reagent was optimized. Thiocarbonyl chlorins were shown to have a significant bathochromic shift in the absorption and fluorescence bands. The feasibility of functionalizing the thiocarbonyl group at the macrocycle periphery by formation of a Pt(II) metal complex in the chemotherapeutic agent cisplatin was shown. The chemical stability of the resulting conjugate in aqueous solution was studied, and it was found to possess a high cytotoxic activity against sarcoma S37 tumor cells that results from the combined photodynamic and chemotherapeutic effect on these cells.

## 1. Introduction

**Figure 1:**
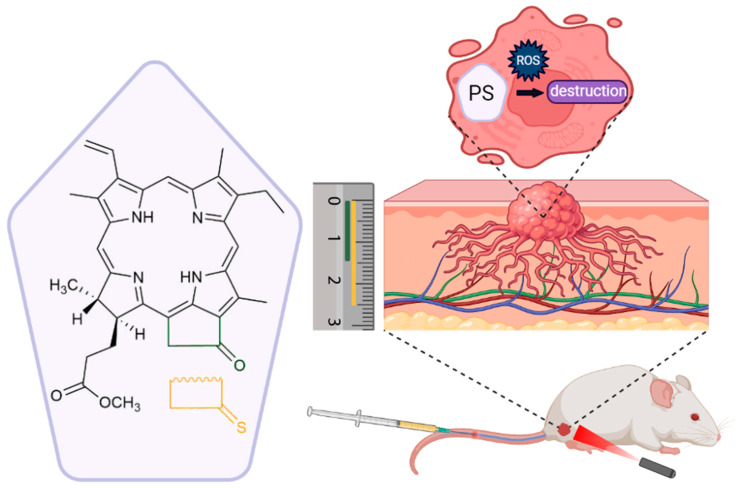
A chlorin and its application in experimental oncology (created with Biorender).

Photodynamic therapy (PDT) is a relatively new and promising method for the treatment of malignant tumors. Despite the achievements in cancer treatment by standard methods (surgery, radiation therapy, chemotherapy), certain problems, such as resistance of tumor cells, metastasis, and, as a consequence, recurrence of the disease, have not been solved yet [1,2,3,4]. PDT is advantageous over traditional treatment methods because it is localized (the cytotoxic effect develops in the irradiated area only), making it possible to overcome multiple drug resistance (MDR) of tumor cells, activate the body’s immune response, and prevent surgical risks and severe systemic complications, and the cost of treatment is low [5,6,7]. The method is based on the ability of a PS to be selectively accumulated in the tumor tissue due to specific features of tumor cells and then generate reactive oxygen species (ROS) under laser light with a certain wavelength. These species have a cytotoxic effect on the tumor nodule cells. The membranes, organelles, and various biomolecules in tumor cells are destroyed during a PDT session due to the main cytotoxic agent, i.e., singlet oxygen. The efficiency of the method depends on many factors, including the PS type, tumor histogenesis, parameters and mode of light irradiation, degree of tumor oxygenation, etc. (Figure 1) [8,9,10,11].

The structure of natural chlorins allows their directed functionalization by incorporation of heteroatoms or biomolecules with desired properties on the periphery of the macrocycle [12,13]. Modified amino acids that are widely used in anticancer therapy are examples of such biomolecules [14]. Previously, our research team obtained derivatives of natural chlorins with various sulfur-containing molecules, including cysteine, lipoic acid, glutathione, etc. [15,16,17,18,19].

Trung Thanh Nguyen et al. suggested a method for the thionation of 2-hydroxyacetophenone by treatment of a solution of the latter in ethanol with gaseous H_2_S and HCl. To prevent the trimerization or oligomerization of the reaction products due to formation of disulfide bonds, the substrate concentration, reaction temperature, intensity, and duration of feeding the reacting gases were controlled. However, the low rate of this reaction limits its use in fine organic synthesis [20].

An alternative thionation method involves the use of phosphorus pentasulfide (P_4_S_10_). For example, a thioketone was obtained in a high yield (70%) from a framework ketone, pentacyclo[5.4.0.02,6.03,10.05,8]undecan-8-one, using P_4_S_10_ in pyridine. However, the capabilities of this approach are limited by the need to handle phosphorus pentasulfide under anhydrous conditions because of the flammability and hydrolysis of the latter in the presence of moisture [21].

A substituted dithiophosphethane known as Lawesson’s reagent is the best-known thionating agent for ketones, amides, and lactams. It is widely used in the chemistry of tetrapyrrole compounds to convert carbonyl derivatives into thio derivatives [22,23]. Such a modification of porphyrins results in a bathochromic shift in the long-wave band in the electron absorption spectrum in comparison with oxygen-containing analogues [24,25].

For example, replacement of the carbonyl oxygen atom with sulfur in a synthetic bacteriochlorin molecule shifts the absorption maximum to the near-infrared spectrum region, while the stability of the resulting dye decreases [26].

It was shown previously that in a group of natural chlorins, pyropheophorbide *a* derivative containing the thiocarbonyl functional group had a greater photodynamic activity than the oxo derivative [27].

Moreover, it should be taken into account that thioketones are susceptible to hydrolysis in physiological media [28,29]; therefore, metal complexes with a coordination center on the macrocycle periphery are often obtained to stabilize them. M.A. Carvalho et al. synthesized a porphyrin metal complex containing a thiocarbonyl group chelating the Pt(II) cation. In subsequent works of this research team, they created nanoribbons based on the porphyrin metal complexes obtained that manifested a batochromic shift in all absorption maxima [30,31].

The purpose of this work was to obtain sulfur-containing chlorins and Pt(II) complexes based thereon and to study their spectral and biological properties.

## 2. Results and Discussion

### 2.1. Synthesis Thiocarbonyl Derivatives

Natural chlorins with absorption maxima in the 660–760 nm range, low dark cytotoxicity, and functional groups on the periphery of the macrocycle were chosen as the starting compounds.

**Scheme 1:**
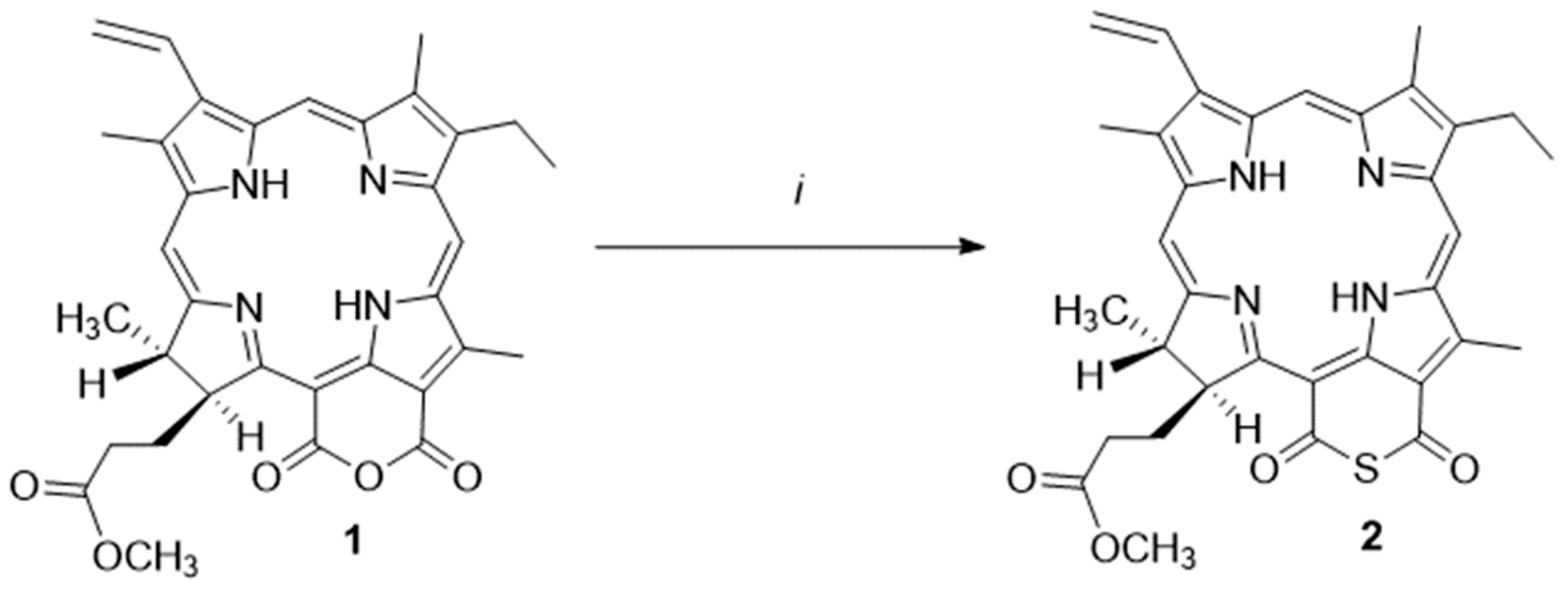
Reagents and conditions. *i*: Lawesson’s reagent, toluene, Et_3_N, 110 °C, 24 h, inert argon atmosphere.

Previously, our team developed synthetic approaches to cyclic chlorin p_6_ thioanhydride (**2**), which was found to be a promising photosensitizer [32,33]. Because the method for the synthesis of thioanhydrides from phthalic and 1,8-naphthalic anhydrides and their derivatives [34,35,36] using thionating Lawesson’s reagent is known in the literature, in this work, we performed a similar reaction with purpurin-18 (**1**) under basic conditions (Scheme 1).

It was found that the reaction occurred ambiguously due to the presence of three carbonyl groups, and as a result, the target thioanhydride (**2**) was obtained in a low yield (15%).

**Scheme 2:**
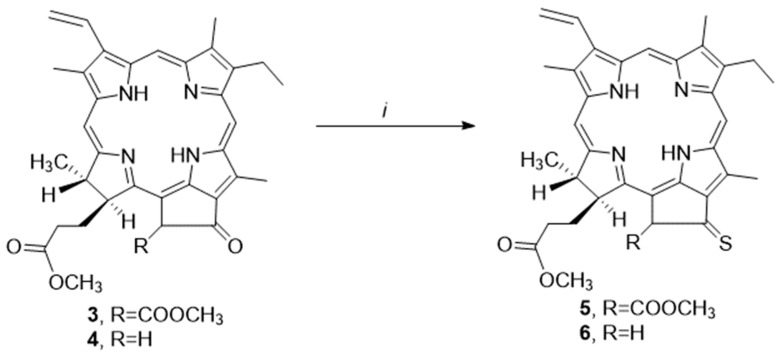
Reagents and conditions. *i*: Lawesson’s reagent; the reaction conditions are listed in Table 1.

To make the thionation reaction with Lawesson’s reagent occur regioselectively, pheophorbide *a* and pyropheophorbide *a* obtained by the technique developed previously from *Spirulina platensis* [37] and containing an isolated carbonyl group in exocycle E were taken as the starting compounds. The starting reagents were converted to thiocarbonyl derivatives using Lawesson’s reagent in toluene, but the yields of the reactions were rather low, i.e., 36 and 39%, respectively (Scheme 2).

To increase the yield, the reaction was performed at various temperatures from 0 °C to 110 °C and with organic bases such as pyridine, DIPEA in toluene, Et_3_N in toluene, and K_2_CO_3_ in acetonitrile (Table 1).

Based on the data in Table 1, the optimum yields of thioketones (**5**) and (**6**) of 73% and 82%, respectively, are achieved using toluene and Et_3_N at 35 °C. Under these conditions, the conversion of the starting pigments occurs at a high rate in 45 min.

**Scheme 3:**
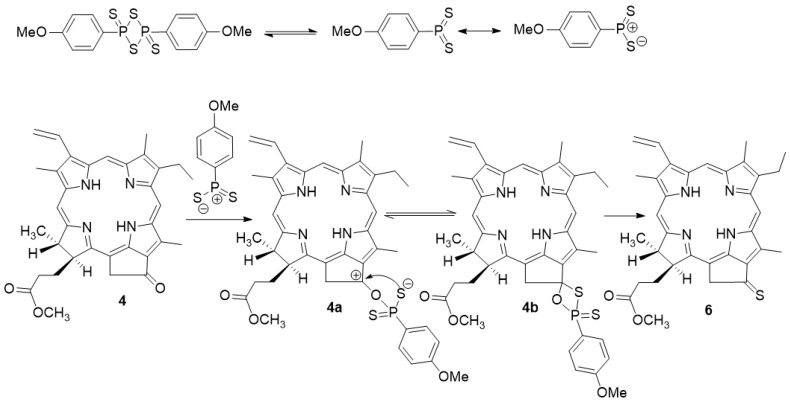
Scheme for the interaction of Lawesson’s reagent with ketones of pyropheophorbide *a*.

The assumed mechanism of this reaction consists of a number of stages (Scheme 3). The first one involves the decomposition of Lawesson’s reagent into two nucleophilic dithiophosphine ylides and addition of one of these at the oxygen atom of the keto group in the cyclopentanone ring to yield thiaoxaphosphetane (**4a**). At the last stage, the C-O bond in compound (**4b**) is broken and a thiocarbonyl group C=S is formed in the target product (**6**).

A possible thionation mechanism was already suggested previously for compounds (**5**) and (**6**) [27], and our research team succeeded in an indirect confirmation of the mechanism of this reaction by high-performance chromatography–mass spectroscopy that detected a molecular ion of the intermediate compound (**4b**) (Figure S1).

The structures of the target chlorins (**5**) and (**6**) were confirmed by ^1^H and ^13^C NMR spectra. The ^13^C NMR spectrum manifests a downfield shift in the signal of the 13^1^ carbon atom, indicating that the oxygen atom is replaced by a sulfur atom (Figure S5).

The purity of the compounds obtained was confirmed by high-resolution chromatography–mass spectrometry spectra, where characteristic molecular ions of pheophorbide *a* 13^1^-thioketone (**5**) and pyropheophorbide *a* 13^1^-thioketone (**6**) were recorded (Figure S1).

### 2.2. UV-Vis Spectroscopy, Fluorescence, and Stability

**Figure 2:**
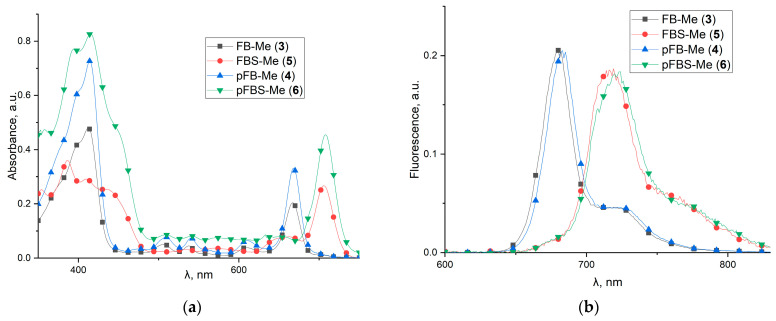
Absorption (**a**) and fluorescence (**b**) electronic spectra: pheophorbide *a* (**3**), pyropheophorbide *a* (**4**), pheophorbide *a* 13^1^-thioketone (**5**), pyropheophorbide *a* 13^1^-thioketone (**6**). The absorption and fluorescence spectra were obtained at the same concentration using dichloromethane as the solvent (created with Origin).

Because the pigments noted above are photoactive compounds, it was important to determine the effect of the sulfur atom on their spectral characteristics. The UV-Vis electronic absorption spectra of the thiocarbonyl derivatives manifest a considerable bathochromic shift in the long-wave absorption maximum by 40 nm in comparison with the original compounds, where it is 666 nm and 668 nm for compounds (**3**) and (**4**), respectively. The Q_2_ band of compound (**5**) in the EAS is at 706 nm, and that of compound (**6**) is at 708 nm (Figure 2a). The fluorescence spectra were recorded under excitation into the absorption band at 550 nm, with fluorescence maxima at 719 nm and 723 nm for thiocarbonyl derivatives (**5**) and (**6**), which are 40 nm higher than those of the parent compounds (**3**) and (**4**), having the fluorescence maxima at 680 nm and 684 nm, respectively (Figure 2b).

The thiocarbonyl derivative of pyropheophorbide *a* (**6**) has a number of advantages over pheophorbide *a* thioketone (**5**), including higher synthetic yields, better stability in storage, etc., so the former was chosen for the further studies.

The synthesis of the water-soluble pigment form (**6**) revealed its instability in aqueous solutions, so we studied this fact in particular detail. The stability of pyropheophorbide *a* 13^1^-thioketone (**6**) in 4% aqueous micellar solution of Kolliphor ELP over time was studied at a neutral pH by monitoring the variation in the Q_2_ band intensity in the absorption spectra of the sample.

**Figure 3:**
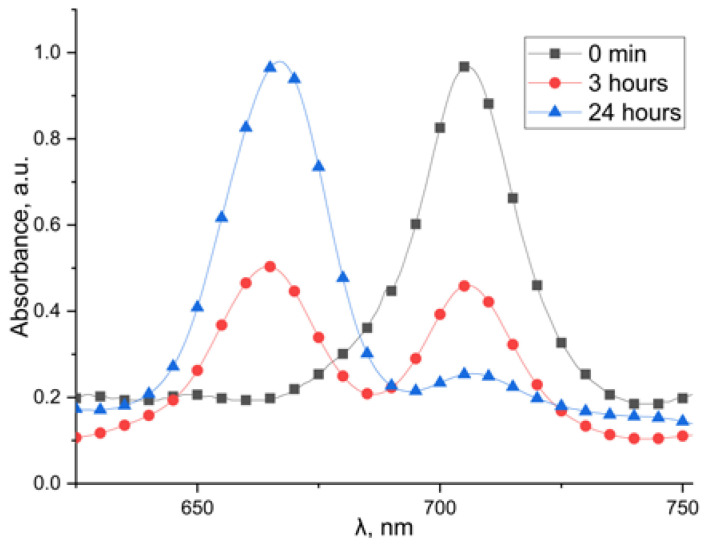
Electronic absorption spectra of compound (**6**) in 4% micellar solution of Kolliphor ELP recorded versus time (created with Origin).

After 3 h, the Q_2_ band of thioketone (**6**) decreased by more than 50%, and after 24 h, the thioketone was completely converted to the original pyropheophorbide *a* (**4**) (Figure 3).

We suggest that the oxidation of thioketones to ketones is associated with PS self-photosensitization. Photosensitized oxidation of thioketones produces ketones as isolated photoproducts, and the proposed reaction mechanism involves the formation of intramolecular “SO” and the release of atomic oxygen to form [38,39].

### 2.3. Synthesis Platinum Complex (II)

To stabilize the thiocarbonyl group in chlorin (**6**), a Pt(II) metal complex was obtained by attaching a cisplatin residue to the periphery of the macrocycle through the sulfur atom. The choice of the well-known chemotherapeutic agent is not accidental, as studies performed abroad and in our laboratory show that combining moieties implementing photodynamic and chemotherapeutic effects on the tumor in a single molecule increases the PDT efficiency [4,5,6,40].

**Scheme 4:**
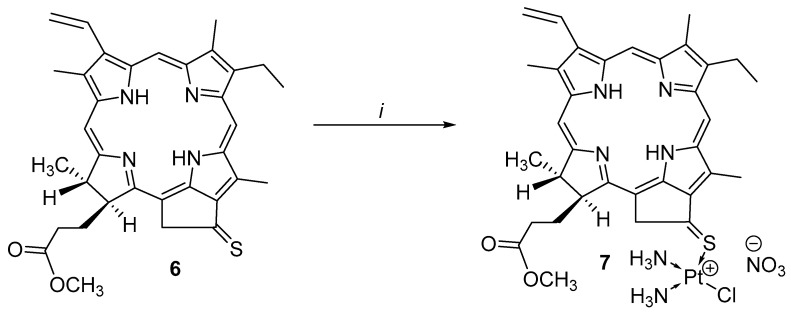
Reagents and conditions. *i*: cisplatin, AgNO_3_, DMF.

Previously, metal complexes of chlorins with platinum were obtained by our team by incorporating chelators based on nicotinic acid, isonicotinic acid, terpyridine, etc., into the PS structure [41]. It is known that apart from the nitrogen atom, the sulfur atom also tends to chelate d-metals due to transfer of unshared electron pairs of the chalcogen to the free orbitals of the metal. To perform metalation, the cisplatin derivative was brought into the reaction with pyropheophorbide *a* 13^1^-thioketone (**6**) (Scheme 4).

Compound **7** was isolated by preparative TLC and characterized by physicochemical methods, including NMR spectroscopy, mass spectrometry, and elemental analysis.

### 2.4. Stability of Platinum Complexes (II)

**Figure 4:**
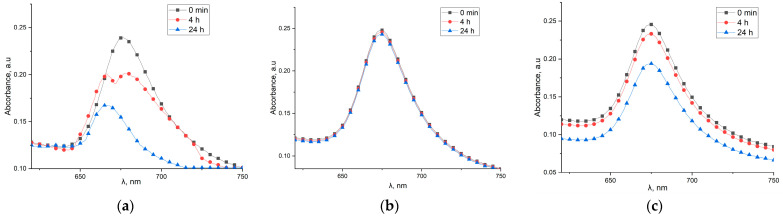
Electronic absorption spectra of pigment (**7**) in 4% micellar solutions of Kolliphor ELP at various pH values vs. time: (**a**) pH = 4, (**b**) pH = 7, (**c**) pH = 9 (created with Origin).

At the next step, we studied the chemical stability of complex **7** obtained in Kolliphor ELP aqueous micellar solutions vs. time at various pH values based on the variation in the intensity of the *Q_y_* band in the EAS of the samples (Figure 4).

At neutral pH = 7, compound **7** remained stable for 24 h, while in an alkaline solution (pH = 9), a precipitate formed and the intensity of the *Q_y_* absorption band decreased. In an acid solution (pH = 4), a hypsochromic shift in the *Q_y_* band to the 666 nm region occurred due to elimination of the platinum complex and hydrolysis of the thiocarbonyl derivative to the methyl ester of pyropheophorbide *a* (**4**), whose structure was confirmed by ^1^H NMR and mass spectra.

The chemical stability of compounds **4** and **7** was also studied in the lysate of S37 sarcoma cells (pH = 7.8), as well as in the cell culture medium at different pH values. The stability was assessed by the change in the intensity of the *Q_y_* bands as described above. The results of the study are shown in Table 2.

At neutral and basic pH, both compounds remained stable in the lysate of S37 sarcoma cells and in the cell culture medium, while a significant decrease in the intensity of the *Q_y_* band was observed in acidic medium.

### 2.5. Biological Studies In Vitro

To estimate the combined effect (photoinduced and chemocytotoxic) of compound (**4**) and platinum complex (**7**), a two-step experiment was designed: incubation of cells with PS for 4 h and irradiation and subsequent incubation for 48 or 72 h followed by estimation of cell survival by the MTT assay. The results of these studies are presented in Table 3.

Incubation of these dyes with S37 sarcoma cells for 72 h showed that the compound comprising the Pt(II) complex (**7**) had a pronounced cytotoxic effect: its IC_50_ value was 271 ± 7 nM, whereas the parent compound (**4**) had no cytotoxic effect on cells.

**Figure 5:**
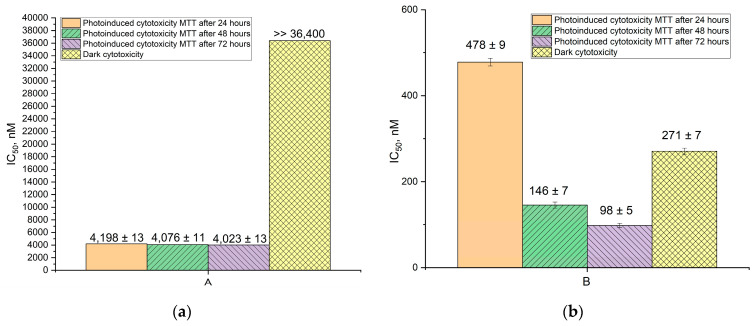
Photoinduced and cytotoxic activity. (**a**) pyropheophorbide *a* methyl ester (**4**): orange sans stripes—4 h PS incubation with cells, irradiation at 10 J/cm^2^, MTT after 24 h; green stripes to the left—4 h PS incubation with cells, irradiation at 10 J/cm^2^, MTT after 48 h; purple with stripes to the right—4 h PS incubation with cells, irradiation at 10 J/cm^2^, MTT after 72 h; yellow with stripes to the right and left—72 h incubation of compounds with cells, MTT. (**b**) chlorin-based Pt(II) metal complex (**7**): orange sans stripes—4 h PS incubation with cells, irradiation at 10 J/cm^2^, MTT after 24 h; green stripes to the left—4 h PS incubation with cells, irradiation at 10 J/cm^2^, MTT after 48 h; purple with stripes to the right—4 h PS incubation with cells, irradiation at 10 J/cm^2^, MTT after 72 h; yellow with stripes to the right and left—72 h incubation of compounds with cells, MTT (created with Origin).

A pronounced additive effect was identified for the PS with a platinum residue (**7**), whose photocytotoxicity increased 5-fold on increasing the incubation time after irradiation from 24 h (IC_50_ 478 ± 9 nM) to 72 h (IC_50_ 98 ± 5 nM) (Figure 5).

No additive effect was observed for pyropheophorbide *a* methyl ester (**4**), and its photoinduced activity was 8.8 times lower than that of the platinum complex.

It was found that the metal-free compound (**4**) does not have a pronounced cytotoxicity without light irradiation. Moreover, platinum complex 7, even without light irradiation, has a higher cytotoxicity with respect to S-37 cells than compound **4** upon irradiation. From this, it follows that the introduction of a fragment of cisplatin changes the effect of the drug in the dark. We hypothesize that this mechanism involves the binding of the cisplatin fragment to DNA [42]. In the absence of irradiation, the chlorin, which has proven tumor tropism [43], acts as a transporter that delivers the cisplatin fragment to the tumor cell, while when irradiated with light, there is a combined (photocytotoxic and chemotherapeutic) effect, leading to an increase in the cytotoxic effect.

Thus, a high cytotoxic activity against S37 sarcoma tumor cells was detected for the platinum complex (**7**) due to the combined photodynamic and chemotherapeutic effect on the latter.

## 3. Materials and Methods

All the solvents used in this work were prepared by conventional techniques as described elsewhere. Column chromatography was performed on silica gel 40/60 (Merck, Darmstadt, Germany). Preparative thin-layer chromatography (TLC) was performed on glass plates with silica gel 60 (Merck, Darmstadt, Germany). Analytical TLC was performed on aluminum plates with Silica gel 60 F254 with a fluorescent probe (Merck, Darmstadt, Germany).

Absorption and fluorescence spectra were recorded on Shimadzu UV1800 UV/VIS (Shimadzu, Duisburg, Germany) and Shimadzu RF-5301 (Shimadzu, Duisburg, Germany) spectrophotometers in quartz cells (0.4 × 1.0 cm) with an optical path length of 1 cm (spectral slit width 1 nm), using dichloromethane, acetone, and water as the solvents. Absorption spectra were recorded in the range of 350–750 nm. Fluorescence spectra were recorded in the range of 600–800 nm (excitation wavelength 550 nm), using dichloromethane, acetone, and water as the solvents. The fluorescence quantum yield was determined by the relative method. A solution of purpurin-18 in toluene was used as the standard [44]. Both ^1^H and ^13^C NMR spectra were recorded on a Bruker DPX 300 spectrometer (Bruker, Alzenau, Germany). NMR samples were prepared in d_6_-acetone and CDCl_3_. Matrix-assisted laser desorption/ionization (MALDI) mass spectra were recorded on a Bruker Ultraflex TOF/TOF spectrometer (Bruker, Alzenau, Germany), with 2,5-dihydroxybenzoic acid as the matrix. Elemental analysis was performed on a Vario Micro Cube analyzer (Elementar, Berlin, Germany).

Extraction of chlorophyll *a* from *Spirulina platensis* and synthesis of purpurin-18 (**1**), pheophorbide *a* (**3**), pyropheophorbide *a* (**4**), and cyclic thioanhydride of chlorin p_6_ (**2**) were carried out as described previously [32,37,45,46].

### 3.1. Synthesis

#### 3.1.1. Cyclic Thioanhydride of Chlorin p_6_ (**2**)

Purpurine-18 17^3^-methyl ester (**1**) (173 mmol, 100 mg) was dissolved in 10 mL of toluene and 1 mL of triethylamine. Then, 140 mg (346 mmol) of Lawesson’s reagent was added. The reaction was carried out under reflux for 24 h with stirring in an inert argon atmosphere. After that, the solvent was removed by evaporation on a rotary evaporator. The compound was purified by column chromatography in the system: (CH_2_Cl_2_/CH_3_OH, 60/1, *v*/*v*). The yield of the target compound (**2**) was 15 mg (15%).

#### 3.1.2. Pheophorbide a 13^1^-Thioketone (**5**)

Pheophorbide *a* (**3**) (82.4 mmol, 50 mg) was dissolved in 5 mL of toluene. Then, 67 mg (165 mmol) of Lawesson’s reagent and 100 μL of triethylamine was added. The reaction was carried out at room temperature in an inert argon atmosphere with stirring for 24 h. The solution was concentrated and purified by preparative TLC (CH_2_Cl_2_/hexane 1/80, *v*/*v*). The yield of the target compound (**5**) amounted to 37 mg (73%).

UV/VIS (CH_2_Cl_2_) λ_max_, nm (ε, M^−1^ sm^−1^): 386 (27,381), 410 (22,072), 706 (20,252).

HRMS *m*/*z* [M+H]^+^ calculated for C_36_H_38_N_4_O_4_S [M+H]: 623.2667. Found: 623.2666.

^1^H NMR (300 MHz, CDCl_3_) *δ* 9.43 (s, 1H, 10-H), 9.25 (s, 1H, 5-H), 8.46 (s, 1H, 20-H), 7.95 (dd, *J* = 17.8, 11.5 Hz, 1H, 3^1^-H), 7.73 (dd, 1H, *J* = 1.4, 18 Hz, 3^2^-H^a^), 7.55 (dd, 1H, *J* = 1.4, 13 Hz, 3^2^-H^b^), 6,58 (s, 1H, 13^2^-H), 6.25 (m, 1H, 18-H), 5.39 (m, 1H, 17-H), 3,85 (s, 3H, 13^4^-CH_3_), 3.75 (s, 3H, 12^1^-CH_3_), 3,60 (m, 5H, 8^1^-H, 17^4^-CH_3_), 3.37 (s, 3H, 2^1^-CH_3_), 3.18 (s, 3H 7^1^-CH_3_), 2.33 (m, 4H, 17^1^-H, 17^2^-H), 1.82 (d, *J* = 7.3 Hz, 3H, 18^1^-H), 1.68 (t, *J* = 7.7 Hz, 3H, 8^2^-H), 0.69 (s, 1H, NH), −1.11 (s, 1H, NH).

^13^C NMR (75 MHz, CDCl_3_) *δ* 219.54, 173.24, 173.10, 169.76, 167.72, 160.20, 156.19, 151.47, 149.47, 146.59, 145.21, 142.63, 138.93, 136.30, 132.38, 125.38, 122.85, 107.82, 104.77, 97.61, 93.21, 74.64, 74.27, 52.85, 51.64, 51.09, 50.01, 38.65, 31.87, 29.65, 28.86, 19.32, 17.28, 12.00, 11.62, 11.11, 0.97.

#### 3.1.3. Pyropheophorbide a 13^1^-Thioketone (**6**)

Pyropheophorbide *a* (**4**) (91 mmol, 50 mg) was dissolved in 5 mL of toluene. Then, 74 mg (182 mmol) of Lawesson’s reagent and 100 μL of triethylamine were added. The reaction was carried out at room temperature in an inert argon atmosphere with stirring for 4 h. The solution was concentrated, and the target compound was isolated by preparative TLC (CH_2_Cl_2_/hexane 1/80, *v*/*v*). The yield of compound (**6**) was 41 mg (82%).

UV-Vis λ_max_ nm (relative intensities of peaks): 386 (23,673), 410 (19,175), 708 (17,518) (1:0.81:0.74).

HRMS *m*/*z* [M+H]^+^ calculated for C_34_H_36_N_4_O_2_S [M+H]: 565.2613. Found: 565.2611.

^1^H NMR (300 MHz, CDCl_3_) *δ* 9.42 (s, 1H, 10-H), 9.27 (s, 1H, 5-H), 8.42 (s, 1H, 20-H), 7.96 (dd, *J* = 17.8, 11.5 Hz, 1H, 3^1^-H), 6.21 (m, 4H, 3^2^-H, 13^2^-H), 5.62 (q, *J* = 19.5 Hz, 2H, 8^2^-H), 4.43 (m, 1H, 18-H), 4.25 (m, 2H, 17-H), 3.69 (s, 3H, 12-CH_3_), 3.66 (m, 2H, 8^1^-H), 3.67 (s, 3H, 12-CH_3_), 3.62 (s, 3H, 17^4^-CH_3_), 3.36 (s, 3H, 2-CH_3_), 3.20 (s, 3H, 7-CH_3_), 2.33 (m, 4H, 17^1^-H, 17^2^-H), 1.82 (d, *J* = 18.2 Hz, 3H, 18-CH_3_), 1.69 (t, *J* = 7.7 Hz, 3H, 8^2^-CH_3_), 0.07 (s, 1H, NH), −1.1 (s, 1H, NH).

^13^C NMR (75 MHz, CDCl_3_) *δ* 226.51, 173.39, 172.50, 159.53, 148.65, 145.04, 142.19, 138.78, 136.71, 136.07, 136.01, 129.02, 122.63, 108.81, 104.52, 97.23, 93.16, 77.38, 77.15, 76.95, 76.53, 59.51, 51.66, 49.91, 31.87, 30.94, 29.64, 22.92, 19.36, 17.31, 12.01, 11.59, 11.14, 0.97.

#### 3.1.4. Platinum Complex of Pyropheophorbide a 13^1^-Thioketone (**7**)

Cisplatin (15 mg, 0.05 mmol, 1 equiv.) and silver nitrate (9 mg, 0.05 mmol, 1 equiv.) were dissolved in DMF (2 mL). The mixture was stirred for 24 h at room temperature in the dark. The resulting solution was centrifugated to remove the silver chloride precipitate. The supernatant was added to pyropheophorbide *a* 13^1^-thioketone (**6**) (0.033 mmol), and the mixture was stirred for 48 h at room temperature. The solvent was distilled off under reduced pressure. The residue was purified by preparative TLC (CH_2_Cl_2_/CH_3_OH, 5/1, *v*/*v*).

UV-Vis (4% micellar solutions Kolliphor ELP) λ_max_, nm (relative intensities of peaks): 406 (33,858), 508 (10,500), 675 (12,703).

HRMS m/z [M+H]^+^ calculated for C_34_H_41_ClN_6_O_2_PtS [M+H]: 828.2423. Found: 828.2425.

^1^H NMR (300 MHz, C_3_D_6_O) *δ* 9.40 (s, 1H, 10-H), 9.21 (s, 1H, 5-H), 8.78 (s, 1H, 20-H), 8.00 (m, 1H, 3^1^-H), 6.25 (dd, 1H, *J* = 17.8, 1.5 Hz, 3^2^-H^a^), 6.10 (dd, 1H, *J* = 11.6, 1.5 Hz, 3^2^-H^b^), 4.69 (s, 1H, 15^1^-H), 4.57 (m, 1H, 18-H), 4.32 (m, 1H, 17-H), 3.57 (s, 3H, 17^3^-COOCH_3_), 3.48 (s, 3H, 12-CH_3_), 3.38 (s, 3H, 2-CH_3_), 3.04 (s, 3H, 7-CH_3_), 2.95 (m, 2H, 8^1^-CH_2_), 2.64 (m, H, 17^2^-CH_2_^a^), 2.40 (m, H, 17^1^-CH_2_^a^), 2.25 (m, 17^2^-CH_2_^b^), 2.18 (m, H, 17^1^-CH_2_^b^), 1.81 (d, 3H, *J* = 7.3 Hz, 18-CH_3_), 1.55 (t, 3H, *J* = 7.6 Hz, 18-CH_3_), 0.87 (t, 3H, *J* = 7.8 Hz, 8^2^-CH_3_), 0.14 (s, 1H, NH), −2.11 (s, 1H, NH).

^13^C NMR (75 MHz, C_3_D_6_O) *δ* 195.69, 174.11, 172.97, 162.17, 155.56, 151.44, 145.73, 142.11, 138.52, 136.87, 136.42, 132.64, 131.52, 130.56, 130.21, 128.71, 122.96, 107.45, 104.74, 97.59, 94.50, 52.71, 51.83, 50.89, 32.47, 31.66, 23.55, 23.45, 19.80, 17.84, 14.50, 12.33, 11.92, 11.22.

Elemental analysis for CHNS, calculated for C_34_H_42_ClN_7_O_5_PtS (%): C, 45.82; H, 4.75; N, 11.00; S, 3.60. Found (%): C, 45.83; H, 4.77; N, 11.01; S, 3.59.

### 3.2. Preparation of Micellar Solutions of Compounds (***4***) and (***7***)

A solution of compounds (**4**) or (**7**) in dichloromethane (1–2 mL) was added dropwise with continuous argon bubbling to a freshly prepared 4% aqueous solution of Kolliphor ^®^ ELP (5 mL) heated to 45 °C. Bubbling was continued until strong foaming started. A clear dark-red solution with a concentration of 0.25 mg/mL was obtained, which was then successively filtered through a 0.45 µm PTFE filter and a 0.22 µm PTFE filter. The concentrations of the resulting micellar solutions were monitored spectrophotometrically.

### 3.3. Biological Studies In Vitro

#### 3.3.1. Characterization of Test Systems In Vitro

The S37 murine sarcoma cell line (collection of the N.N. Blokhin Research Institute of Oncology) was used to estimate photoinduced activity and cytotoxicity.

Cells were cultured in DMEM medium (PanEco, Russia) with addition of 2 mM L-glutamine (PanEco, Moscow, Russia) and 10% fetal calf blood serum (FCS) (Corning, New York, NY, USA) in 25 cm^2^ culture vials (Corning, USA) at 37 °C in a humid atmosphere containing 5% carbon dioxide. Upon passivation, the Versene solution (PanEco, Russia) was used to remove the cells from the substrate. For in vitro studies, cells of the 3rd and 4th passages were used with an optimal seeding concentration of 6 × 10^4^ cells/mL.

#### 3.3.2. Preparation of the Substances to Be Studied

The concentrations of the solutions obtained were 0.6 mg/mL for compound (**4**) and 0.1 mg/mL for compound (**7**). All the volumes obtained were filtered through a Millipore membrane filter with a pore size of 0.22 μm. Next, the samples were diluted in 24-well plates (Corning, USA) on the cell-culturing medium. The final concentration of the substances ranged from 32 ng/mL to 20 μg/mL.

#### 3.3.3. Procedure for Studying Photoinduced and Dark Activity In Vitro

To perform the experiments, S37 tumor cells were seeded into 96-well plates in the amount of 6 × 10^3^ cells per well in a 100 μL volume. Exposure was performed in the exponential phase of cell growth 24 h after seeding. Solutions of compounds in the complete culture medium were added to the wells at final concentrations of 32 ng/mL to 20 μg/mL, in triplicate. The time of cell incubation with compounds before irradiation was 4 h. The culture medium was used as the blank. Light exposure was performed with a halogen lamp through a KS-10 broadband filter (λ > 620 nm). The light dose was 10 J/cm^2^. After light exposure, the plates with cells were placed in a CO_2_ incubator for 24, 48, or 72 h.

To estimate cytotoxicity, cells were incubated with the substances for 72 h under dark conditions in a CO_2_ incubator.

Cell survival was estimated using the colorimetric MTT test (24 and 72 h after exposure), which is based on the ability of dehydrogenases in living cells to convert yellow water-soluble 3-(4-5-dimethyl-tetrazolyl-2)-2,5-diphenyltetrazolium bromide to blue crystals of formazan, whose amount is measured spectrophotometrically.

Before the test, cells were washed from cytotoxic agents. Then, 0.5% solution of 3-(4,5-dimethylthiazolyl-2)-2,5-diphenyltetrazolium bromide was added to the wells and the system was incubated for 3 h under standard conditions. After incubation, the medium with the MTT-reagent was removed and formazan crystals were dissolved for 10 min with dimethyl sulfoxide. Optical density was measured on a Multiscan FC multichannel plate photometer (Thermo scientific) at 550 nm.

The growth inhibition (GI) of cells in a culture was calculated using the following formula:

GI(%) = [OD_k_ − OD_o_/OD_k_)] × 100%,

where OD_o_ is the optical density of formazan in the test wells and OD_k_ is the optical density of formazan in the control wells.

Inhibition of cell growth in culture by more than 50% (IC_50_) was considered a biologically significant effect. Quantitative parameters were calculated using the results of three independent tests.

## 4. Conclusions

In this work, methyl esters of chlorin p_6_ thioanhydride (**2**) and thiocarbonyl derivatives of pheophorbide *a* (**5**) and pyropheophorbide *a* (**6**) were obtained using Lawesson’s reagent. We indirectly confirmed the mechanism of thionation of compound (**4**) by identifying an intermediate compound by means of high-performance chromatography–mass spectrometry. The spectral properties of thiocarbonyl derivatives of pheophorbide *a* (**5**) and pyropheophorbide *a* (**6**) were studied. A considerable bathochromic shift in the long-wave absorption and fluorescence maxima by about 40 nm compared to the carbonyl precursors (**3**) and (**4**) was shown.

The chelating capabilities of the thiocarbonyl group at the periphery of the macrocycle were shown by formation of a Pt(II) metal complex in cisplatin, a chemotherapeutic agent. The chemical stability in aqueous solutions of the conjugate of pyropheophorbide with cisplatin was studied, and the high cytotoxic activity against sarcoma S37 tumor cells due to the combined photodynamic and chemotherapeutic effects was shown.

## Figures and Tables

**Table 1:** Thionation conditions.

Thioketone	Solvent	Temperature	Time	Yield
**5**	toluene	0 °C	48 h	24%
	toluene	20 °C	4 h	36%
	toluene	35 °C	1 h	56%
	toluene	70 °C	30 min	43%
	toluene	110 °C	30 min	19%
	pyridine	35 °C	30 min	61%
	toluene, DIPEA	35 °C	45 min	67%
	toluene, Et_3_N	35 °C	45 min	73%
	K_2_CO_3_ in acetonitrile	82 °C	60 min	32%
**6**	toluene	0 °C	48 h	27%
	toluene	20 °C	4 h	39%
	toluene	35 °C	1 h	62%
	toluene	70 °C	30 min	54%
	toluene	110 °C	30 min	21%
	pyridine	35 °C	30 min	69%
	toluene, DIPEA	35 °C	45 min	77%
	toluene, Et_3_N	35 °C	45 min	82%
	K_2_CO_3_ in acetonitrile	82 °C	60 min	33%

**Table 2:** Intensity of the absorption maxima of compounds (**4**) and (**7**) under cell culture medium and cell lysate S37.

			Compound 7		Compound 4	
Time	Incubation Medium	pH	Intensity at 675 nm	Content, %	Intensity at 666 nm	Content, %
0 min	Cell culture medium	4	0.91299	100	0.95382	100
		7	0.91300		0.95379	
		9	0.91298		0.95381	
	Cell lysate S37	-	0.91302		0.95380	
4 h	Cell culture medium	4	0.22824	25	0.66767	70
		7	0.88561	97	0.88702	93
		9	0.79429	87	0.81073	85
	Cell lysate S37	-	0.81257	89	0.86795	91
24 h	Cell culture medium	4	0.15520	17	0.57229	60
		7	0.85822	94	0.82980	87
		9	0.68473	75	0.74397	78
	Cell lysate S37	-	0.76692	84	0.78212	82
72 h	Cell culture medium	4	0.10042	11	0.55321	58
		7	0.83297	91	0.75349	79
		9	0.52770	65	0.70582	73
	Cell lysate S37	-	0.72127	79	0.72489	76

**Table 3:** Photoinduced and cytotoxic activity of pyropheophorbide a methyl ester (**4**) and chlorin-based Pt(II) metal complex (**7**). S37 cell culture.

Compound	IC_50_, nM			
	Photoinduced cytotoxicity	Combined cytotoxicity		Dark cytotoxicity
	4 h PS incubation with cells Irradiation at 10 J/cm^2^, MTT after 24 h	4 h PS incubation with cells Irradiation at 10 J/cm^2^, MTT after 48 h	4 h PS incubation with cells Irradiation at 10 J/cm^2^, MTT after 72 h	72 h incubation of compounds with cells, MTT
(**4**)	4198 ± 13 nM	4076 ± 11 nM	4023 ± 13 nM	>>36,400 nM
(**7**)	478 ± 9 nM	146 ± 7 nM	98 ± 5 nM	271 ± 7 nM
Cisplatin	-	-	-	17,185 nM ± 15 nM

## Data Availability

The data presented in this study are available from the authors.

## Supplementary Materials

The following supporting information can be downloaded at: https://www.mdpi.com/article/10.3390/molecules28104215/s1, Figure S1: Mass chromatogram of the reaction mass, target compound mass time 11.27 min, m/z [M+H]^+^ = 751.2538; Figure S2: Mass chromatogram of compounds (**5**) and (**6**): (I) chromatogram of 13^1^-thioketone pheophorbide *a* (**5**) retention time 11.43 min; (II) HRMS spectrum of 13^1^-thioketone pheophorbide *a* (**5**); (III) chromatogram of 13^1^-thioketone pyropheophorbide *a* (**6**) retention time 12.39 min; (IV) HRMS spectrum of 13^1^-thioketone pyropheophorbide *a* (**6**); Figure S3: ^1^H NMR spectrum of compound **5**; Figure S4: ^1^H NMR spectrum of compound **6**; Figure S5: ^13^C NMR spectra. (**a**): pheophorbide *a* (**3**)–red, pheophorbide *a* thioketone (**5**)–blue; (**b**): pyropheophorbide *a* (**4**)–red, pyropheophorbide *a* thioketone (**6**)–green (created with MestRenova); Figure S6: ^1^H NMR spectrum of compound **7**; Figure S7: ^13^C NMR spectrum of compound **7**; Figure S8: HRMS spectrum of compound **7**.
