# Supplementary material for: Thiocarbonyl Derivatives of Natural Chlorins: Synthesis Using Lawesson’s Reagent and a Study of Their Properties
Source: Molecules. 2023 May 20;28(10):4215. doi: 10.3390/molecules28104215 (PMC10222512; doi:10.3390/molecules28104215)
Supplement: Supplementary file 1 [file molecules-28-04215-s001.zip › molecules-2390771-supplementary.pdf]

Supporting information

Thiocarbonyl derivatives of natural chlorins: synthesis using the Lawesson's reagent and a study of their properties

Viktor Pogorilyy, Petr Ostroverkhov, Valeria Efimova, Ekaterina Plotnikova, Olga Bezborodova, Ekaterina Diachkova, Yuriy Vasil'ev, Andrei Pankratov, and Mikhail Grin

CONTENTS

Instrumental procedures of analysis 2

Instrumental procedures of analysis

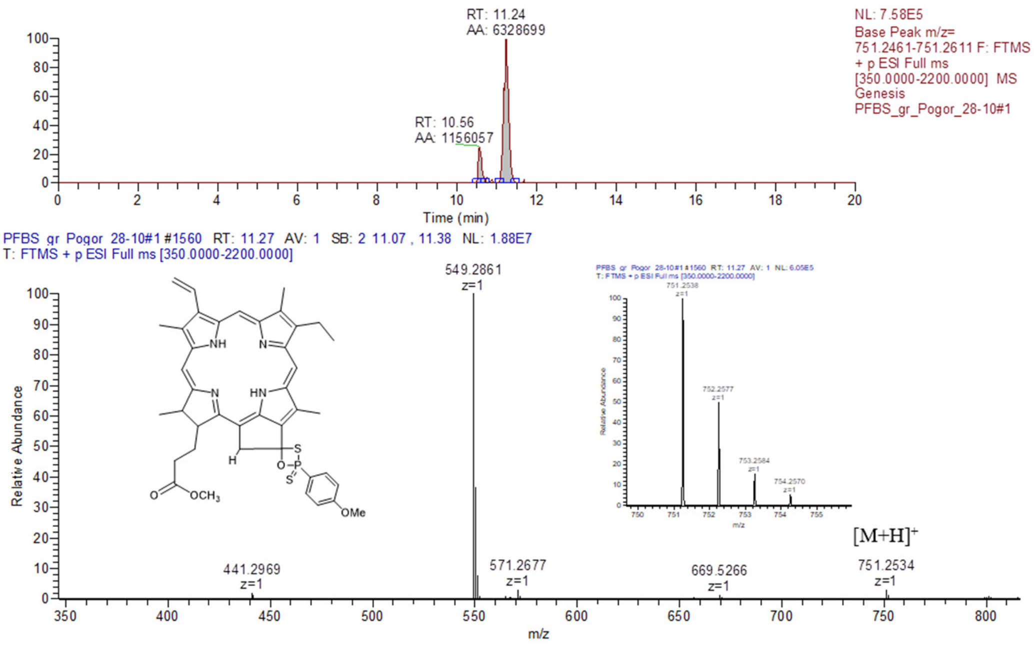

Figure S1. Mass chromatogram of the reaction mass, target compound mass time 11.27 min, m/z $[M+H]^+$ = 751.2534.

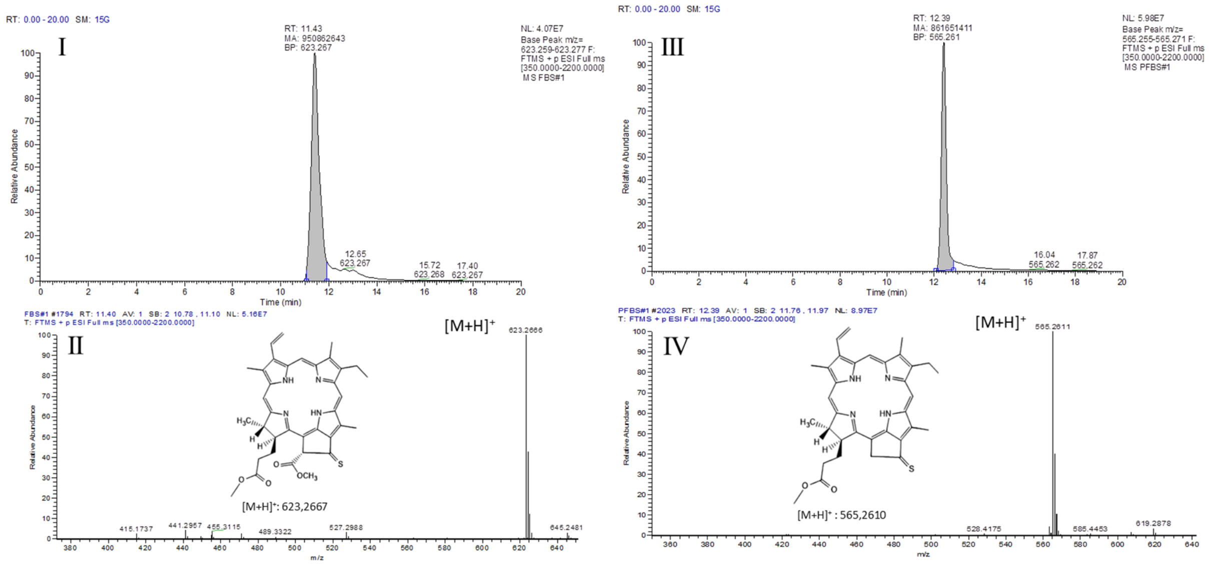

Figure S2. Mass chromatogram of compounds (**5**) and (**6**): (I) chromatogram of 13^1-thioketone pheophorbide *a* (**5**) retention time 11.43 min; (II) HRMS spectrum of 13^1-thioketone pheophorbide *a* (**5**); (III) chromatogram of 13^1-thioketone pyropheophorbide *a* (**6**) retention time 12.39 min; (IV) HRMS spectrum of 13^1-thioketone pyropheophorbide *a* (**6**).

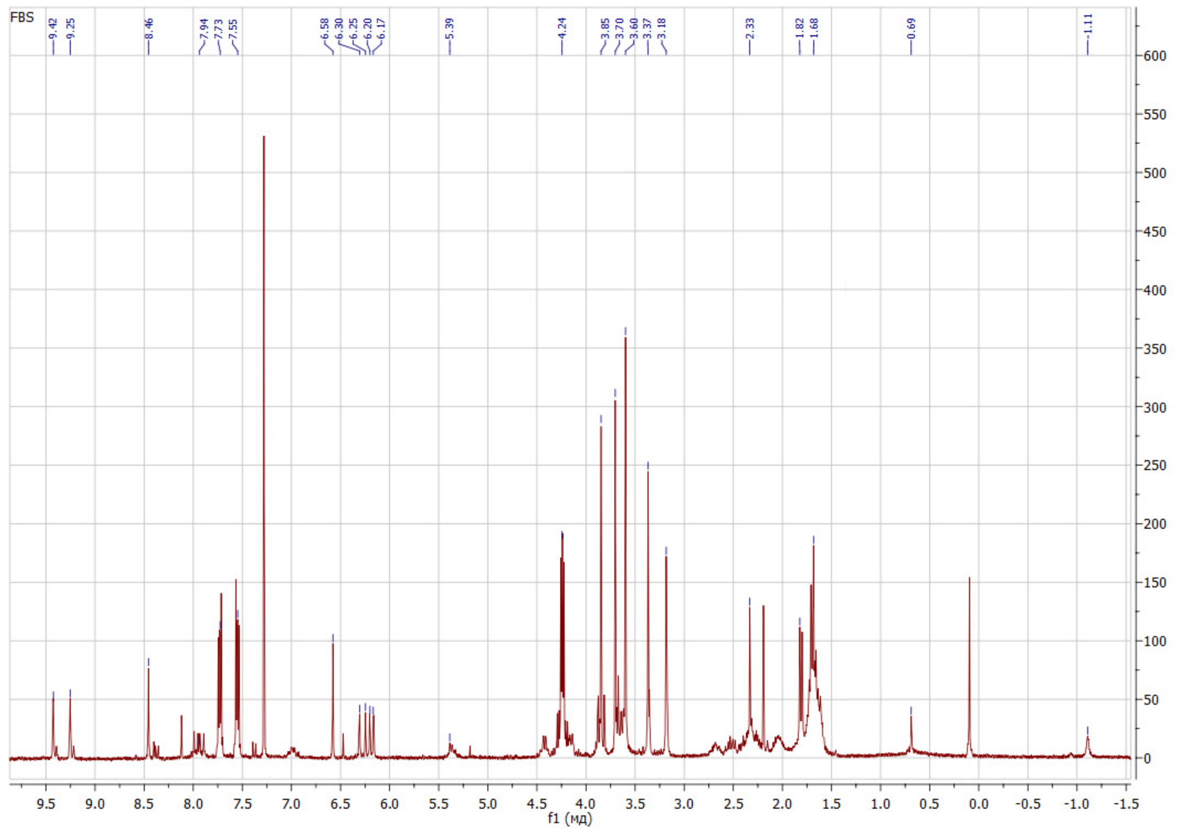

Figure S3. ^{1}H NMR spectrum of compound **5**.

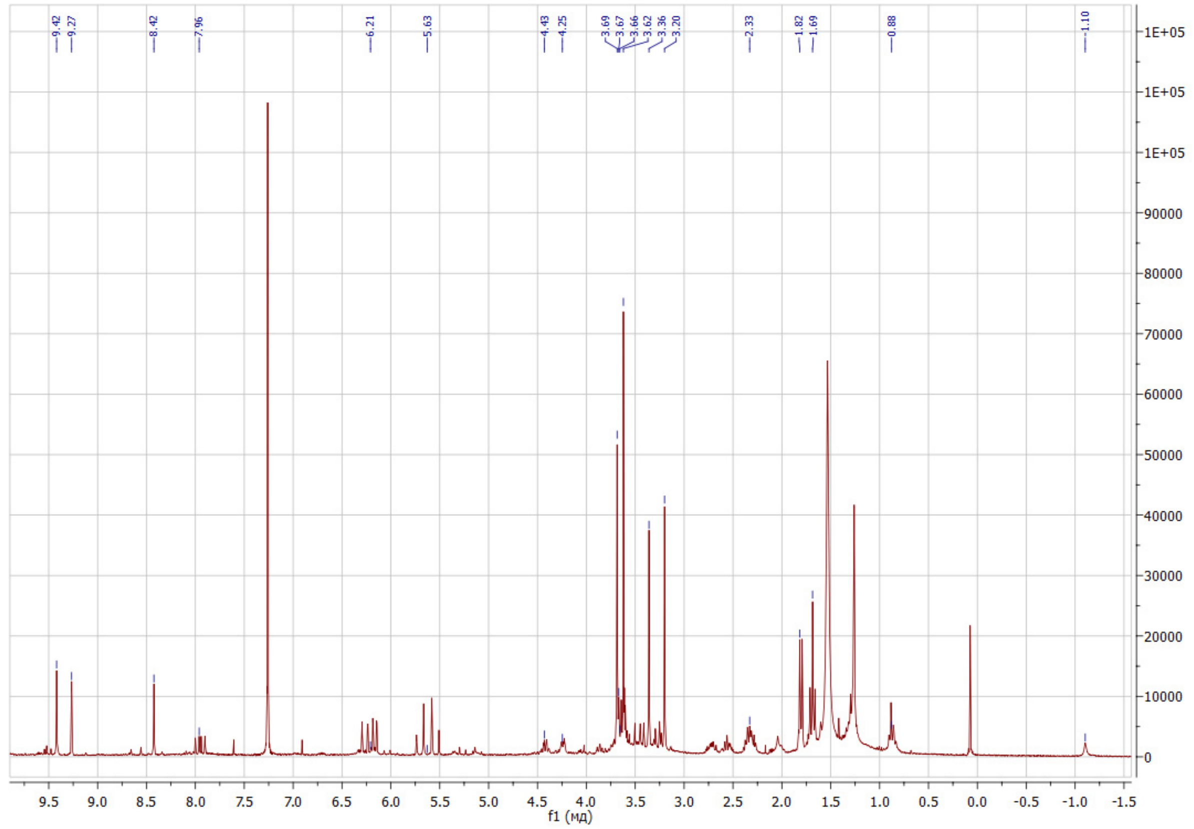

Figure S4. ^{1}H NMR spectrum of compound **6**.

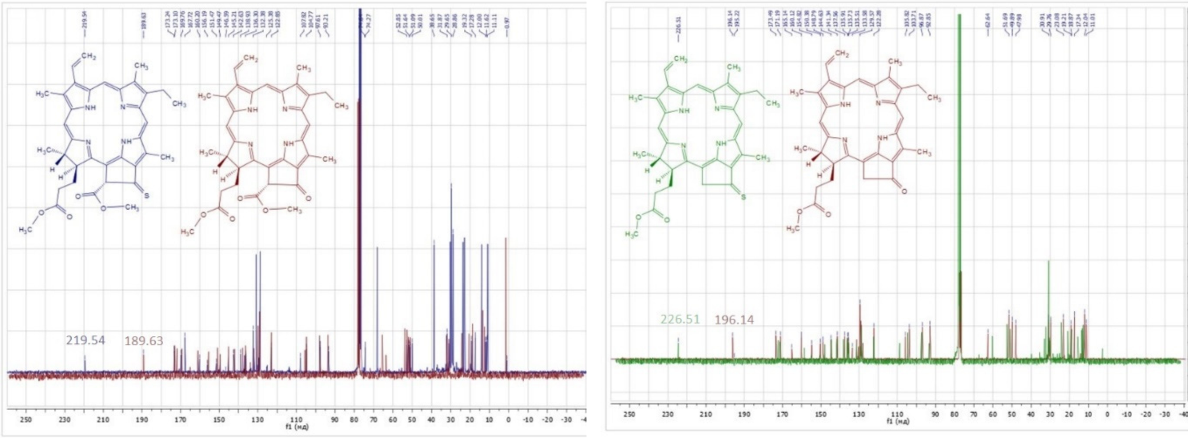

(**a**) (**b**)

Figure S5. ^{13}C NMR spectra. (**a**): pheophorbide *a* (**3**) – red, pheophorbide *a* thioketone (**5**) – blue; (**b**): pyropheophorbide *a* (**4**) – red, pyropheophorbide *a* thioketone (**6**) – green (created with MestRenova)

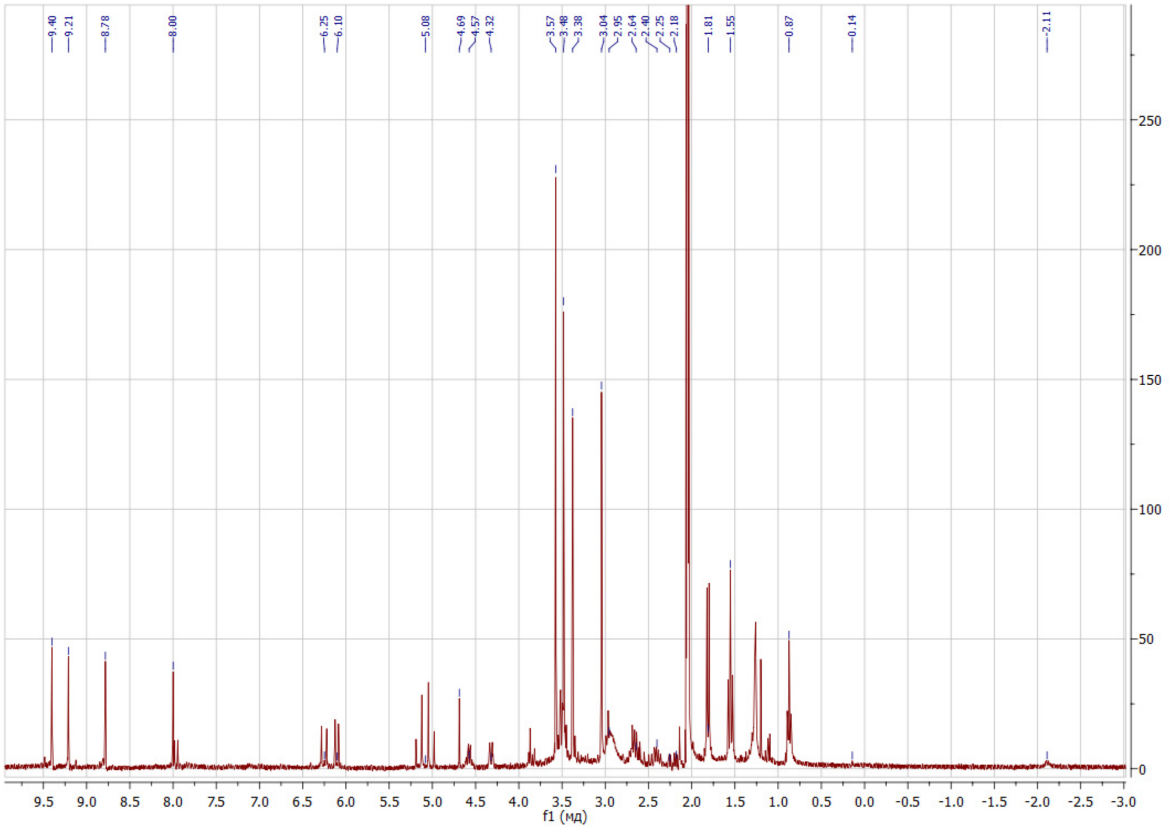

Figure S6. ^{1}H NMR spectrum of compound **7**.

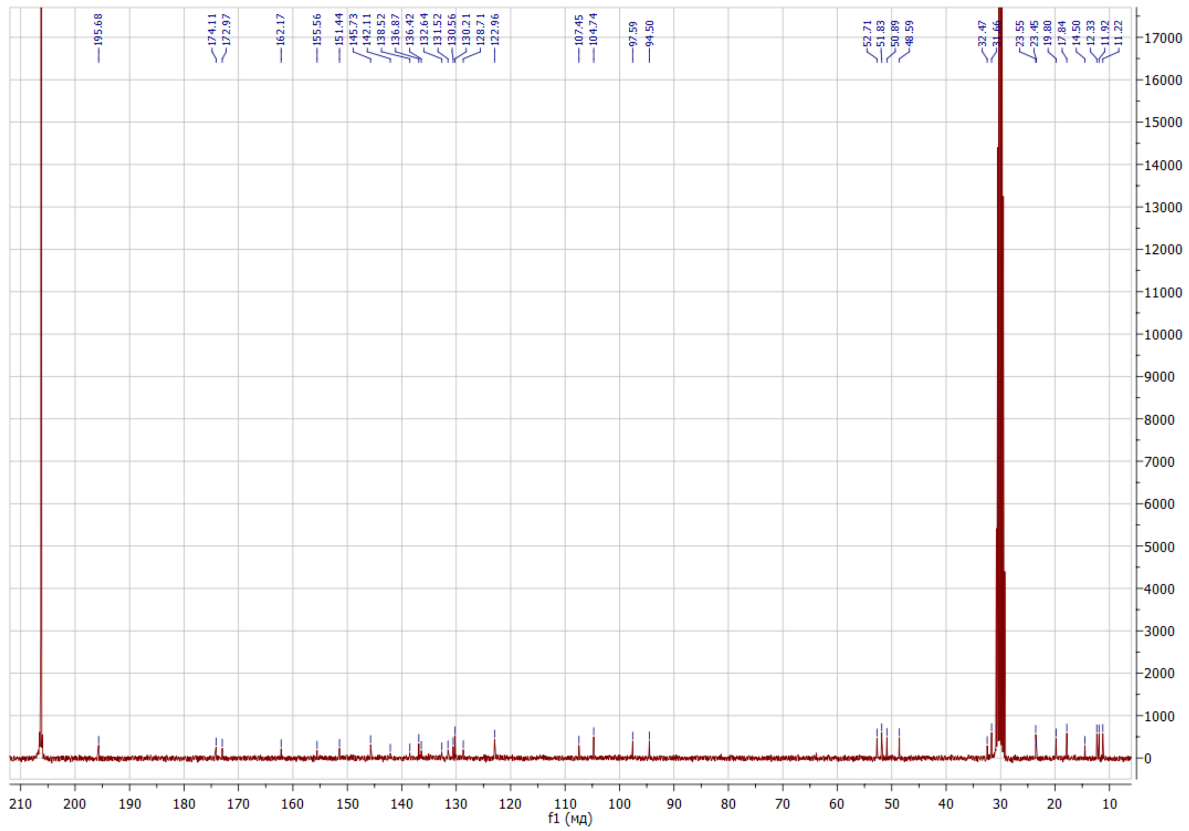

Figure S7. ^{13}C NMR spectrum of compound **7**.

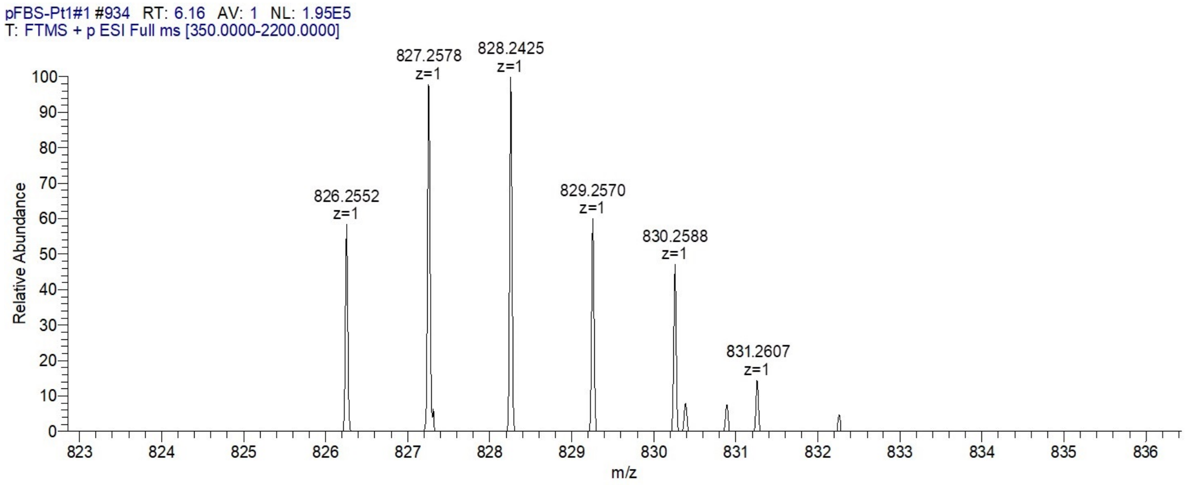

Figure S8. HRMS spectrum of compound **7**.
